# Nature and Regulation of Protein Folding on the Ribosome

**DOI:** 10.1016/j.tibs.2019.06.008

**Published:** 2019-11

**Authors:** Christopher A. Waudby, Christopher M. Dobson, John Christodoulou

**Affiliations:** 1 Institute of Structural and Molecular Biology, University College London and Birkbeck College, London, UK; 2 Centre for Misfolding Diseases, Department of Chemistry, University of Cambridge, Cambridge, UK

**Keywords:** co-translational folding, free energy landscape, molecular chaperones, protein synthesis, translation kinetics

## Abstract

Co-translational protein folding is an essential process by which cells ensure the safe and efficient production and assembly of new proteins in their functional native states following biosynthesis on the ribosome. In this review, we describe recent progress in probing the changes during protein synthesis of the free energy landscapes that underlie co-translational folding and discuss the critical coupling between these landscapes and the rate of translation that ultimately determines the success or otherwise of the folding process. Recent developments have revealed a variety of mechanisms by which both folding and translation can be modulated or regulated, and we discuss how these effects are utilised by the cell to optimise the outcome of protein biosynthesis.

## Highlights

Many proteins can begin to fold and begin to assemble their quaternary structure during biosynthesis on the ribosome.
Co-translational folding is a nonequilibrium process, the outcome of which is dependent on the interplay between the rate of protein folding and the rate of translation by the ribosome.
Co-translational folding takes place across a series of free energy landscapes, encoded in the amino acid sequence, that depend on the length of the nascent chain and can be modulated by interactions with the ribosome surface and with molecular chaperones.
The kinetics of protein synthesis can vary in a sequence-specific manner encoded in the genome.
Through a combination of these effects, co-translational folding pathways may be tuned to maximise the efficiency of protein biosynthesis.

## How Do Proteins Fold within the Cell?

Protein folding is essential to life: not only is the efficient formation of stable native structures central to biological function, but misfolding and aggregation are implicated in a wide range of pathological disorders [1]. In the half century since Cyrus Levinthal’s seminal observation that protein folding cannot be a random search across conformational space [102], great strides have been made in understanding the basic principles that underlie this process [2]. The description of folding as a diffusive search across a **free energy landscape** (see Glossary) has proved to be an important development both to provide a conceptual view of the way that proteins can fold in finite times and to provide insight into the manner in which the process of folding is encoded in the sequence 3, 4. However, the majority of experimental studies of protein folding have focused on the reversible folding of a relatively small number of relatively small (≤100 amino acids; aa) proteins, typically following chemical or thermal denaturation [5]. In contrast, the folding of larger proteins, often comprising multiple domains or subdomains, is more likely to be initiated during synthesis on the ribosome [6]. Our progress in understanding the folding of such systems will be the focus of this review.

**Figure 1:**
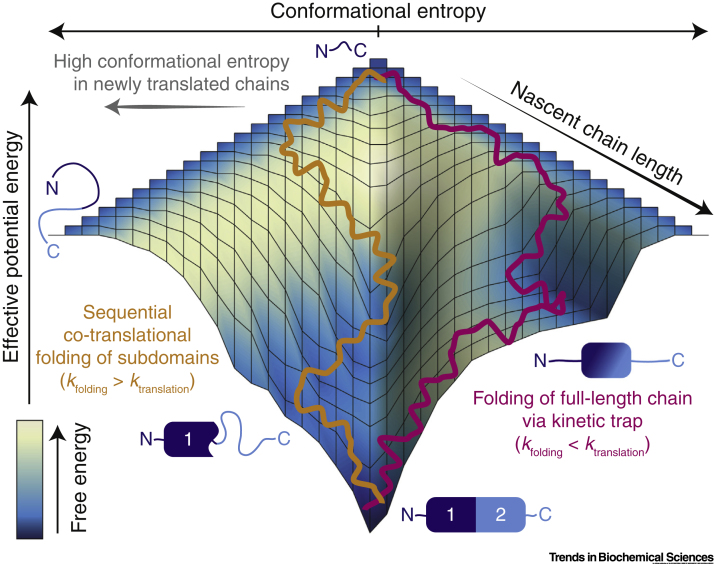
3D Schematic Illustration of the Length-Dependent Free Energy Landscape of a Hypothetical Protein, Comprising Two Subdomains in Its Native State Emerging from the Ribosome During Biosynthesis. The width of the surface represents the conformational entropy of the chain, while the depth represents the effective potential energy (averaged over solvent interactions) [94]. The surface of the landscape is shaded to indicate the total free energy (at each point), which reflects the competition between favourable conformational entropy or potential energy. In this depiction, the left and right halves of the surface are used to illustrate different regions of the free energy landscape; the left and right edges both represent disordered conformations and can be considered identical. An intermediate free energy minimum is shown on the left hand side that corresponds to the folding of the N-terminal subdomain prior to the complete synthesis of the C-terminal subdomain. A second free energy minimum emerges at longer chain lengths (depicted on the right hand side), corresponding to a kinetically trapped, misfolded intermediate; this is illustrated here by interactions between the N-terminal subdomain and the partially translated C-terminal subdomain. Two trajectories are shown corresponding to two co-translational folding scenarios, depending on the relative rates of folding and translation, that is, rapid folding relative to translation (orange, *k*_folding_ > *k*_translation_), and slow folding relative to translation (magenta, *k*_translation_ > *k*_folding_).

As protein biosynthesis takes place within the cell, **nascent polypeptide chains (NCs)** are gradually extruded from the ribosomal **exit tunnel**, making it possible for folding to be initiated at the N terminus and to proceed in a vectorial manner before the C terminus has fully emerged from the ribosome 7, 8, 9. This process of **co-translational folding** cannot simply be described by a single free energy landscape, for the conformational space accessible to the NC expands with its length, and intermediate states may become favoured or disfavoured as it emerges. Therefore, the co-translational landscape must instead be conceptualised as a nested series of length-dependent free energy landscapes spanning increasingly large conformational spaces (Figure 1) 7, 10. In this review, we discuss progress on the experimental characterisation of these landscapes, which also provides a framework to understand recently identified mechanisms through which co-translational folding may be modulated, through passive and perhaps stochastic processes, or regulated, through active interventions in the folding process. Although the experimental studies discussed here have largely been carried out on the prokaryotic ribosome, the underlying physical principles are likely to be similar for eukaryotic systems (Box 1).

Box 1 **Experimental Characterisation of Free Energy Landscapes Associated with Co-translational Protein Folding**

Several experimental strategies have been developed to observe co-translational folding occurring in real- time, largely based on fluorescence measurements of synchronised ribosomes in bulk [97], or single-molecule force spectroscopy [98]. However, identifying and characterising the various states populated along such pathways, and the transitions between them, is a challenging task [99]. Therefore, a key complementary approach to studying the length-dependent free energy landscapes associated with co-translational protein folding is the analysis of translationally-arrested RNCs to provide equilibrium ‘snapshots’ of co-translational folding occurring at defined NC lengths [100]. This approach can also be supplemented by the study of N-terminal protein fragments to create a ribosome-free model of length-dependent free energy landscapes, enabling perturbations arising from ribosomal attachment to be discerned and more fully understood (Figure IA) [14].

A wide variety of techniques have been applied to probe folding within RNCs, including biochemical methods based on covalent modification, proteolysis, or disulfide bond formation (Figure IA) 19, 26, 37, 39, 76, and folding-induced force release of arrest peptides 24, 33. At a structural level, while cryo-electron microscopy is able to probe the early folding of NCs within the exit tunnel and the vestibule region 24, 33, 81, it has not yet been possible to characterise NCs beyond the vestibule due to their dynamic properties. In this regard, solution-state NMR spectroscopy is a powerful complementary technique that can resolve more flexible regions of NCs that have emerged beyond the exit tunnel. In addition to determining the populations of folded or unfolded states (Figure IA), NMR resonances provide hundreds of residue-specific probes of structure, dynamics, and ribosome interactions (Figure IB), allowing a detailed structural characterisation of states populated along the folding landscape (Figure IC) [37]. Single-molecule force spectroscopy can also be a powerful probe both of folded and misfolded populations, and at present has a unique ability to characterise the kinetics of folding within RNCs, and the effects of ribosome–NC or intra-NC interactions on apparent folding rates (Figure ID) 36, 65, 88.

Finally, an increasingly powerful accompaniment to experimental investigations is the use of ‘coarse-grained’ molecular dynamics simulations to develop *in silico* models of co-translational folding 42, 101. These have proved to be powerful in analysing, for example, the potential for tertiary structure formation within the exit tunnel, the length dependence of the free energy landscape of the nascent chain (Figure IE) [101], and the generation of mechanical force at the PTC due to folding of the NC [25]. Computational approaches have also been important for understanding the various effects that arise from the interplay of translation and folding kinetics, particularly through the creation of Markov models to integrate these two processes into unified descriptions of co-translational folding pathways 14, 16.

**Figure I:**
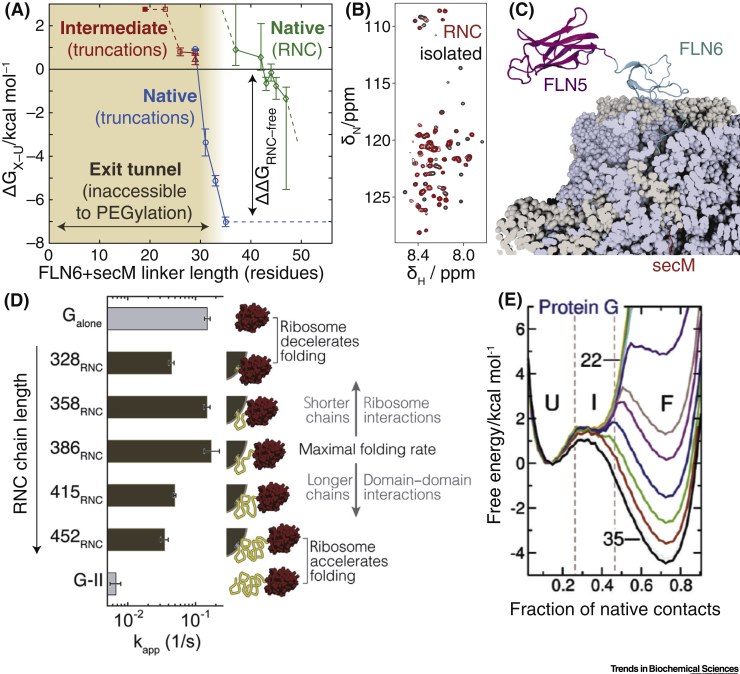
Experimental and Computational Characterisation of Co-translational Folding. (A) NMR and biochemical characterisation of length-dependent folding in an FLN5+6 ribosome–nascent chain complex (RNC) system, showing the extent of the exit tunnel (shaded yellow) measured by the susceptibility of a cysteine residue in an unfolded RNC to PEGylation (resulting from a covalent reaction with a 5 kDa polyethylene glycol maleimide), and free energies for folding of the tethered FLN5 domain (green) determined via an analysis of NMR resonance intensities [37]. Free energies for folding are also shown for isolated native and intermediate states (associated with the isomerisation of a native-state *cis* proline) determined using a C-terminal truncation approach and indicate a substantial destabilisation of the native state on the ribosome 14, 37. (B) The ^1^H,^15^N NMR correlation spectrum of an FLN5+31 RNC, overlaid with that of an isolated unfolded reference, reveals residue-specific broadenings associated with interactions between the ribosome surface and the disordered nascent polypeptide chain (NC) [37]. (C) Snapshot from an ensemble structure of the FLN5+110 RNC system (comprising the FLN5 domain attached to a 110 amino acid linker corresponding to the subsequent FLN6 domain and the secM arrest peptide), determined using molecular dynamics simulations restrained using experimental values of NMR chemical shifts [37]. (D) Apparent folding rate of the G domain of EF-G, measured for isolated G and G-II domains and for varying lengths of G-II RNCs, through repeated force-ramp cycles using optical tweezers [88]. (E) Computational modelling of length-dependent free energy landscapes using coarse-grained molecular dynamics simulations, showing in this case the progressive stabilisation of the folded state ‘F’ of protein G from linker lengths of 22–35 residues [101]. Reprinted (adapted), with permission, from [101]. Abbreviation: PPM. Parts per million.

Alt-text: Box 1

## Describing Co-translational Protein Folding Using Free Energy Landscapes

The nested free energy landscape picture immediately highlights some novel aspects of co-translational folding relative to the folding of a full-length isolated chain. The volume of conformational space (proportional to the width of the folding surface in Figure 1) will in general be smaller for shorter polypeptide chain lengths; this feature is expected to result in more rapid folding relative to larger sequences, although the folding kinetics of individual systems will also depend on other factors such as the complexity of the fold [11]. As translation (synthesis of the NC) increases the conformational space accessible to the NC, the newly emerged portion of the NC is most likely, at least initially, to be in a disordered conformation. The effect of translation is therefore to increase the conformational entropy of the system. This process competes directly with the diffusive search for low energy folded states, and therefore in any consideration of co-translational folding processes it is essential to compare the rate of folding (diffusion across the landscape, which may occur on timescales from microseconds to hours depending on the size and complexity of the fold [11]) with that of translation (typically occurring with rates of 1–20 aa/s [12], although this may be modulated or regulated as described further below).

The effect of the relative rate of translation versus folding is illustrated in Figure 1. If translation is rapid (*k*_translation_ > *k*_folding_), then folding will be initiated from a disordered state in the full-length polypeptide chain and the risk of forming kinetically trapped and potentially misfolded intermediates is increased (Figure 1, magenta trajectory). Such misfolded states have been detected in the folding of tandem repeat proteins and indeed may be formed transiently in more general cases of multidomain protein folding [13]. However, if folding is rapid relative to translation (*k*_folding_ > *k*_translation_), then this misfolded state is unlikely to be populated as the majority of polypeptides will fold in a sequential manner via the N-terminal domain (Figure 1, orange trajectory). Thus, it is clearly desirable in this situation that the rate of translation should be reduced relative to that of folding in order that the co-translational folding process can most effectively assist in the efficient synthesis of fully folded proteins, and a simple kinetic model has been proposed for the quantitative analysis of such cases [14]. However, in other situations, more rapid translation may also be desirable to increase translational efficiency and fidelity in core residues [15], or to minimise the exposure of misfolding-probe segments [16].

Experimentally, translation rates may be modulated by a number of factors, which will be discussed further below, and it is expected that these rates should be tuned according to the particular details of the co-translational folding process. There is now clear evidence that co-translational folding and translation kinetics are under evolutionary selection 17, 18, and that disruption of such tuning can lead to misfolding and impaired protein synthesis 19, 20, 21, 22, 23.

## The Ribosome Can Modulate Co-translational Folding Pathways

**Figure 2:**
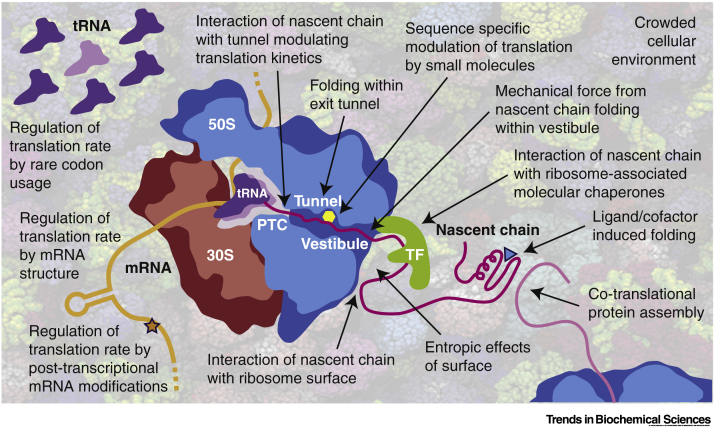
Pathways through Which the Folding of NCs May Be Modulated or Regulated. The background image shows a snapshot from a Brownian dynamics simulation of the bacterial cytosol [95]. Abbreviations: NC, nascent polypeptide chain; PTC, peptidyl-transferase centre; TF, trigger factor.

From a large number of studies, including those discussed above, it is clear that many proteins can fold, or fold partially, during translation, along pathways that in some cases differ from those observed in isolated domains 24, 25, 26. While simple secondary structure elements such as α helices may form within the confines of the ribosomal exit tunnel [27], the acquisition of tertiary structure occurs only upon reaching the **exit tunnel vestibule**. This onset of co-translational folding is dependent upon domain size and stability [28] and may be modulated by the shape of the ribosome exit tunnel [29]. However, it is increasingly apparent that the ribosome is not just a passive spectator during this process but can be an active participant, using a variety of direct and indirect mechanisms to influence the folding of NCs as described below. A key challenge is to understand how the co-translational folding process can be modulated and regulated both by the ribosome and by the broader cellular context (Figure 2).

Within the cell, the crowded environment of the cytosol has been shown to perturb protein stability as a result of the combined effects of excluded volume and weak interactions with cellular macromolecules 30, 31. The presence of cofactors or ligands can also modulate co-translational folding. Thus, for example, ATP binding has been found to promote co-translational folding of the N-terminal nucleotide binding subdomain of human cystic fibrosis transmembrane conductance regulator (CFTR), which in turn facilitates the co-translational folding of other domains of this protein [32]. As a second example, Zn^2+^ binding has been shown to induce the folding of a small zinc-finger domain from the regulatory protein ADR1 within the exit tunnel [33]. The operon structure of multichain proteins can also assist the co-translational folding and assembly of quaternary structure; in an elegant series of experiments, the efficiency of bacterial luciferase heterodimer assembly was shown to be coupled to translation from polycistronic mRNA, ensuring spatial localisation of the nascent subunits [34].

### Interactions with the Ribosome Surface

**Figure 3:**
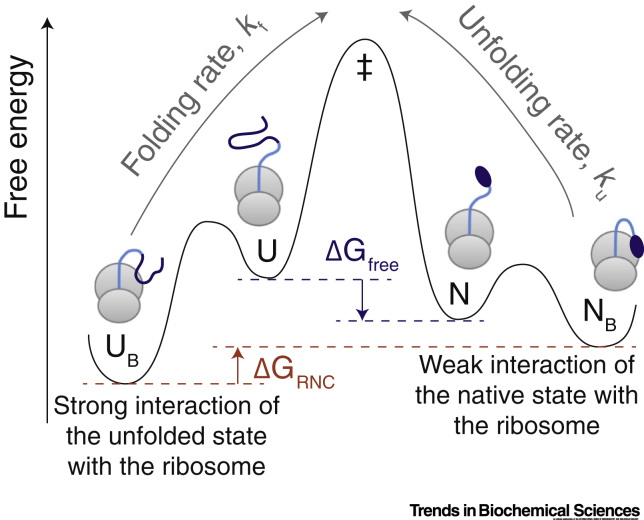
2D Schematic Free Energy Landscape Illustrating the Effect of Interactions between the Nascent Polypeptide Chain (NC) and Ribosome on the Co-translational Folding Process. Unfolded (U) and native (N) states are shown folding through a transition state (‡), and the free energy of folding, in the absence of additional interactions, is ΔG_free_ (indicated by dashed blue lines). However, interactions of unfolded and native states with the ribosome surface to form bound states (U_B_ and N_B_, respectively) may perturb the effective free energy of folding of the ribosome–nascent chain complex (RNC), ΔG_RNC_ (indicated by dashed red lines). A stronger interaction with the ribosome of the unfolded state relative to the native state is illustrated here (depicted by the lower free energy of U_B_ compared with N_B_), the effect of which is (i) that the folding equilibrium is shifted from the native state towards the unfolded state, and (ii) that the rates of folding (*k*_f_) and unfolding (*k*_u_) are both decreased due to the increased free energy barrier heights.

Upon emerging from the ∼100 Å length of the ribosomal exit tunnel and vestibule [35], in most cases the first potential interaction partner to which a nascent polypeptide segment is exposed is the surface of the ribosome itself 36, 37. This surface has traditionally been considered to be chemically inert but able to generate an entropic stabilization of compact species through excluded volume effects [38]. However, interactions of NCs with the ribosome surface can also modulate the co-translational folding process more directly 36, 39. As NCs are covalently attached to the **peptidyl-transferase centre (PTC)** during biosynthesis, regions of the polypeptide chain that have emerged from the exit tunnel are still constrained to be close to the adjacent ribosome surface. This can result in the **effective concentrations** of groups exposed on the ribosome surface reaching values of tens of millimolar orders of magnitude higher than in bulk solution [37]. Consequently, even low-affinity interactions with surface groups may result in strong interactions within ribosome-associated NCs that can thereby significantly perturb the co-translational free energy landscape (Figure 3). Measurements of the thermodynamic stability of a variety of translationally arrested protein domains (attached to linkers of variable lengths) have indicated that unfolded states in particular can be stabilised by up to 2 kcal/mol through interactions with the ribosome [39], and that this increased stability can also be associated with an order of magnitude slower folding kinetics [36]. These effects are reduced at longer linker lengths at which the effective ribosome concentration is reduced and appear to be at least partly electrostatic in nature 36, 39. Together, these observations provide strong evidence that interactions with the ribosome surface can be very significant and can perturb NC folding, imparting an ATP-independent **holdase** functionality to the ribosome surface and so delaying the folding of at least some NCs until later in the translation process [36].

The quantification and characterisation of such ribosome–NC interactions poses challenges to many experimental techniques, but answers to some central questions are beginning to emerge. In particular, time-resolved fluorescence depolarisation, used to quantify the interaction of a disordered NC with the ribosome surface, showed that the populations of bound species were between 60 and 90%, and that binding could be modulated by mutations altering the NC charge [40]. NMR spectroscopy is also exquisitely sensitive to transient interactions between proteins and high molecular weight systems such as the ribosome, and site-specific changes in resonance line widths have been used to identify and quantify weaker interactions (∼1% binding) in several **ribosome–nascent chain complexes (RNCs)** 37, 41, 42; again revealing a correlation with electrostatic charge, but additionally suggesting a particular role for aromatic residues [42]. It is clear, however, that more information is required in order to understand fully the sequence determinants of these interactions, as well as the key sites of interaction on the ribosome surface itself.

## Regulation of Elongation Kinetics

The rate of translation relative to that of folding is of central importance in defining the outcome of co-translational folding (Figure 1). The average translation rate is ∼20 aa/s in bacteria and ∼5 aa/s in eukaryotes [12], but site-specific variations in the translation rate have been revealed by **ribosome profiling** experiments 43, 44, 45 and single-molecule measurements of single-codon translocation kinetics [46]. These variations are encoded in genomes by a range of mRNA and protein-mediated mechanisms 9, 47, 48.

### mRNA-Mediated Translational Regulation

As a consequence of the degeneracy of the genetic code, mRNA sequences are able to contain information beyond that of the primary polypeptide sequence, and this capacity has been found to encode modulations in the rate of translation through several nonmutually exclusive mechanisms.

First, the rate of translation is limited by the encounter and decoding time for cognate tRNA molecules, which in turn depends on the relevant tRNA concentrations, as well as competition with near-cognate tRNAs and **synonymous mutations** between abundant and rare tRNAs; these have been observed to cause order-of-magnitude changes in the translation rate 49, 50. Such variations can be represented in terms of codon usage bias or codon optimality 9, 19, and ribosome profiling measurements on a proteome-wide scale in yeast and bacteria have found that rare or nonoptimal codons are associated with slower translation kinetics 45, 51. Moreover, codon usage is under evolutionary selection: conserved clusters of rare codons are often associated with the boundaries between folding domains, inducing pauses in translation that can facilitate the folding of preceding domains 17, 19, 52 or, based on an analysis of course-grained molecular dynamics simulations of co-translational folding, with co-translational folding intermediates [18]. Many examples of functional consequences arising from synonymous mutations altering codon usage bias have now been reported, in which perturbations in translation kinetics disrupt the co-translational folding process or direct the products towards alternative misfolded conformations 20, 21, 22, 23, 53. Variations or mutations in the tRNA pool between tissues, or in response to the cellular environment or the onset of disease, will also modulate codon optimality, and if translation kinetics are strongly perturbed this can lead to disruption of proteostasis and disease 54, 55.

Secondly, stable mRNA secondary structure elements may, in a limited number of cases, also slow the rate of translation. *In vitro*, the impact of mRNA structure on translation is clear and has been followed in detail using single-molecule measurements [56]. However, the impact on elongation appears to be less significant *in vivo*, as mRNA tends to be less structured and more dynamic than expected from *in vitro* measurements [57]. Indeed, ribosome profiling in *Escherichia coli* has found that with the exception of a small number of particular sites, structured mRNA regions are not generally associated with reductions in the speed of translation [58].

Finally, an additional layer of regulation may be achieved through post-transcriptional chemical modifications of mRNA, termed the epitranscriptome. A number of chemical modifications are known to be present in bacterial and eukaryotic RNAs, although the precise functions and regulation of these changes are currently poorly understood. For example, *N*^6^-methylation of adenosine is known to occur within coding regions of mRNA transcripts, and codons containing this modified base have been found, using single molecular fluorescence methods, to induce significant delays in the translation process [59]; by contrast, acetylation of cytidine at wobble sites can increase the efficiency of translation [60]. While many details clearly remain to be elucidated, it has been suggested that these modifications may have a role in regulating the coupled co-translational folding process [59].

### NC-Mediated Translational Regulation

Independently of the mRNA-mediated mechanisms discussed above, the NC may also regulate the rate of its own translation by making interactions with the tunnel interior that lead to perturbations in the conformation of the PTC active site. The best characterised of such sequences are **arrest peptides**, such as secM, which act as sensors and translational regulators of gene expression [61]. In the case of secM, high-resolution cryo-electron microscopy, combined with single-molecule measurements of translocation kinetics, has shown that this sequence has a multifaceted stalling mechanism in which NC–ribosome interactions within the exit tunnel perturb the geometry at the PTC active site during multiple stages of the elongation cycle, thus inhibiting both peptide bond formation and translocation 62, 63. These NC–tunnel interactions, and hence stalling, may be disrupted by mechanical forces generated by the motor protein of the translocon [64] or those induced by protein folding in the vestibule of the exit tunnel 33, 65. Indeed, such chemomechanical feedback between NC folding and translocation has been exploited to create sensitive assays of co-translational folding near the exit tunnel, based on detecting the force-induced translation of a reporter sequence following an arrest peptide [66]. Translation may also be modulated or arrested by NC interactions with small molecules within the exit tunnel, which provide an efficient means of regulating protein synthesis in response to intracellular metabolite concentrations [67]. This is also the mechanism of action of the macrolide antibiotics, which bind within the exit tunnel and interact selectively with polypeptide motifs to arrest translation [68].

In addition to the extreme examples represented by arrest peptides, translation kinetics can also be modulated by a variety of other types of NC sequences. For example, positively charged polypeptide sequences can reduce the rate of translation of downstream residues through interactions with the negatively charged surface of the exit tunnel 69, 70, while polyproline sequences can stall translation because the most favourable conformation of these sequences within the confines of the exit tunnel is not compatible with further peptide bond formation [71]. This stalling can be relieved by the elongation factor EF-P [72], or by mechanical forces exerted directly by NC folding [73]. Molecular and quantum mechanical simulations also predict that translation rates can be modulated by mechanical forces transmitted to the PTC, for example, through entropic forces generated from unfolded NCs beyond the exit tunnel [74]. Collectively, these effects suggest the existence of a more general mechanism for the dynamic regulation of translation kinetics. Such a mechanism could allow the folding of an NC to modulate directly the rate of its own synthesis [65] through the real-time feedback between diffusion over the free energy landscape and the rate of change of the length-dependent free energy landscape itself (Figure 1).

## Reshaping Length-Dependent Free Energy Landscapes with Molecular Chaperones

**Figure 4:**
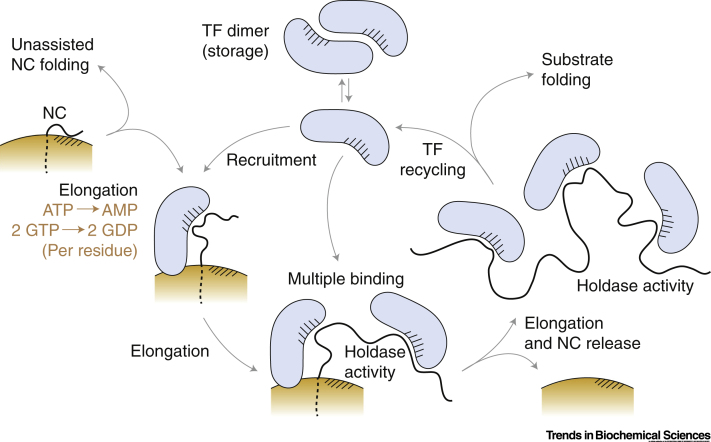
Schematic Cycle of the Chaperone Behaviour of Trigger Factor (TF). Nascent polypeptide chain (NC) binding sites on the ribosome (orange) and TF (grey) are represented by cross-hatching. TF is ATP-independent, but the energy consumption of the associated ribosome elongation process is indicated, corresponding, for each NC residue, to the charging of the incoming aminoacyl tRNA and the activity of elongation factors EF-G and EF-Tu [96].

Small protein domains have, in numerous cases, been observed *in vitro* to fold in isolation, but many more complex domains or larger proteins require the assistance of molecular chaperones to fold efficiently [75]. As discussed above, the ribosome surface may have an intrinsic holdase activity through preferential interactions with disordered states that can inhibit the formation of kinetically trapped intermediates 36, 37, 76. However, the surface can also act as a hub for recruiting other components of quality control systems, and selective ribosome profiling studies are yielding detailed interaction profiles of many such factors 77, 78, 79, providing rich insights into recognition motifs and functions. For example, by correlating interaction profiles of the yeast Hsp70 ribosome-associated holdase Ssb with local translation kinetics, also determined through ribosome profiling, a remarkable covariation of chaperone recruitment and NC translation rate was identified (occurring mainly through intrinsic features of the mRNA and NC), such that holdase-associated NC segments can be rapidly but safely translated [79]. In the remainder of this review we focus on trigger factor (TF), the sole ribosome-associated molecular chaperone in bacteria, which binds near the exit tunnel via the L23 protein in the large ribosomal subunit (Figure 4) 80, 81.

TF exists within the cell as a dimer, within which substrate-binding sites are sequestered to prevent promiscuous interactions with nonclient proteins 82, 83. TF dimers dissociate readily and the resulting monomers interact with ribosomes rapidly and reversibly to scan for substrate NCs; upon locating a NC, dissociation of TF from the RNC is inhibited and RNC binding is therefore stabilised [84]. This occurs with highest affinity in the presence of disordered, hydrophobic NCs with lengths of at least 50 residues 76, 85. This length was found to coincide with the onset of NMR line broadening in an RNC of the intrinsically disordered protein α-synuclein in the presence of TF, suggesting the existence of TF–NC contacts [42]. In addition, molecular modelling indicated that this NC length is also the minimum required to contact TF substrate binding sites identified in a landmark NMR study of isolated TF–substrate interactions [86]. On a global scale, selective ribosome profiling also found that a minimum NC length of 50 residues was required for recruitment of TF to the RNC, although full TF engagement typically only occurs for NC lengths of ∼100 residues and greater 77, 78. This effect provides an opportunity for small proteins with rapid folding rates to reach their native structures without unnecessary sequestration of TF [76], as well as spatially separating the engagement of NCs by TF from recognition by signal recognition particle [78].

Once bound, as translation proceeds TF can remain engaged with substrate NCs for 10–100 s (dependent on affinity), even following dissociation of TF from the ribosome [85]. Indeed, multiple copies of TF may bind to a single NC to delay both folding and misfolding until a sufficient length of NC has been synthesised and has the capacity to fold productively [86]. Through the cumulative interaction of the NC with several individually weak sites (*K*_d_ 10–200 μM), TF can reshape a flat, frustrated free energy landscape into a funnel with an optimal combination of avidity (for an effective *K*_d_ ≤1 μM), plasticity (i.e., recognition of diverse substrates) and reversibility that efficiently suppresses undesirable long-range interactions while allowing rapid dissociation to occur as folding proceeds 86, 87. In the case of the multidomain protein EF-G, TF not only suppressed misfolding between domains, but also protected against the denaturing effect of adjacent unfolded polypeptide chains on previously folded domains [88]. Understanding the interplay of the mechanical force generated by NC folding in the vestibule and TF binding is also of growing interest as TF binding reduces the mechanical force generated by NC folding and transmitted to the PTC [89], which may in turn affect translation kinetics. Furthermore, in the presence of moderate mechanical force (generated by magnetic tweezers in the absence of the ribosome) TF was found in some cases to destabilise the unfolded state resulting in a **foldase** activity [90]. Finally, we note that although TF has no intrinsic ATPase activity, the TF chaperone cycle is coupled to the translocation of the unfolded NC from the exit tunnel. In this sense, TF extracts otherwise unused free energy from the energy-intensive elongation process, and therefore represents a highly efficient and effective mechanism of chaperone activity.

## Concluding Remarks

As described in this review, the process of *de novo* protein folding within the cell can differ significantly from that observed in studies of protein refolding in dilute solutions. Free energy landscapes provide a powerful framework for understanding protein folding, and it is important to understand how these landscapes evolve during the process of protein synthesis, and how they can be sculpted in response to cellular conditions. Ultimately, a deeper understanding of these processes may lead to improved protein expression for biotechnological applications [91]; greater capacity to understand and treat the numerous disorders arising from protein folding deficiencies, protein misfolding, and aggregation [1]; improved understanding of macrolide mechanism and resistance [68]; and the prospect of selectively targeting polypeptide translation for therapeutic purposes using small molecules [92].

Protein biosynthesis and quality control are energy-intensive processes that take place within the context of limited cellular resources, and efficient protein folding has therefore been under strong selective pressure since the earliest stages of the emergence of life [93]. A wide range of mechanisms have been identified that help to ensure that protein biosynthesis within the cell occurs both correctly and efficiently. These mechanisms include passive strategies, such as the evolutionary optimisation of co-translational free energy landscapes and site-specific variations in translation kinetics, and the holdase functionality of the ribosome surface, and active ones, including the intervention of chaperone systems such as TF and ATP-dependent chaperones such as Hsp70, and ultimately the proteasome. The ribosome is a central hub within this quality control network, ultimately providing effective and energy efficient defences against potentially lethal misfolding and aggregation processes [1].

**Figure 5:**
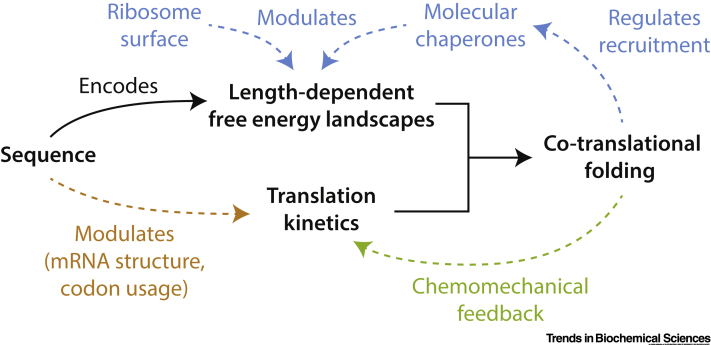
Key Figure. Dynamic Regulation of Co-translational Folding by Modulation of Length-Dependent Free Energy Landscapes and the Rates of Translation, and Recruitment of Molecular Chaperones

In this review we have shown that for co-translational folding within the cell the concept of a static free energy landscape that is appropriate for the folding of a full-length protein in solution must be replaced by a series of length-dependent free energy landscapes, and that the rate of translation between these surfaces may be as critical to the outcome of co-translational folding as the rate of folding itself (Figure 5, Key Figure). Describing the coupling between folding and translation requires the development and application of the appropriate theoretical and experimental tools. The immediate experimental challenges are the development of methods to expand our understanding of the mechanisms by which free energy landscapes, and the kinetics of kinetics may be modulated or regulated (see Outstanding Questions). In particular, a fascinating aspect of protein biosynthesis that has only recently become possible to explore is the development of a molecular understanding of the feedback and interplay of these various mechanisms on co-translational folding processes (Figure 5). The discovery of the coupling between folding and translation processes undoubtedly indicates fertile ground for future research. The further elucidation of these processes, both in prokaryotes and eukaryotes, presents an exciting challenge for the years ahead in the quest to define in molecular detail the way in which information encoded in the genome is converted into biological activity.

Outstanding Questions How does the ribosome surface recognise nascent chains and modulate free energy landscapes? What are the sequence determinants of these interactions within nascent chains, and what are the key sites of interaction on the ribosome surface?

How can experimental investigations of length-dependent free energy landscapes and translation rates be combined to create a complete kinetic and structural description of a nonequilibrium folding process?

How can the sometimes-competing effects of codon usage and mRNA structure, which sometimes generate opposing effects, be brought together to uncover the determinants of the translation rate?

How general is the chemomechanical feedback between co-translational folding and translation kinetics?

How are free energy landscapes and translation kinetics modulated and regulated in eukaryotes compared with prokaryotes?

Alt-text: Outstanding Questions

## Glossary

**Arrest peptide**: short polypeptide sequence, typically with a regulatory function such as the 17-residue secM sequence from the secretion monitor protein, that forms interactions within the ribosome exit tunnel inhibiting further translation or release.
**Co-translational folding**: process through which a protein may begin to acquire its secondary or tertiary structure in a vectorial manner during biosynthesis on the ribosome.
**Effective concentration**: when examining the interaction of two sites linked by a covalent tether (for example, the interaction of NC residues with the ribosome surface), the effective concentration (also known as the local concentration) is the concentration of the binding partner that would be required to achieve the same extent of binding in the absence of the covalent tether.
**Exit tunnel**: passage traversing the large ribosome subunit from the PTC to the ribosome surface, through which NCs emerge during biosynthesis. The exit tunnel is ∼100 Å in length and has a width of 10–20 Å, within which approximately 30–40 residues can be encapsulated.
**Exit tunnel vestibule**: widest point of the exit tunnel, having a width of ∼20 Å and located 20 Å from the ribosome surface, within which simple tertiary structures may begin to form.
**Foldases**: class of molecular chaperones, typically ATP-dependent, which directly assist the folding of an unfolded or misfolded protein into its native structure.
**Free energy landscape**: free energy represents the combination of enthalpic and entropic contributions to protein stability. Free energy landscapes are high dimensional surfaces describing the free energy of a protein as a function of its conformation, but are commonly and usefully represented by low-dimensional projections along macroscopic reaction coordinates, from which discrete states can be identified.
**Holdases**: class of ATP-independent molecular chaperones that interact with unfolded or partially folded states to delay folding and inhibit aggregation and misfolding.
**Nascent polypeptide chain (NC)**: polypeptide chain in the process of being synthesised by the ribosome.
**Peptidyl-transferase centre (PTC)**: site of peptide bond formation and peptide release on the ribosome, located in a cleft in the large subunit at the beginning of the exit tunnel.
**Ribosome–nascent chain complex (RNC)**: ribosome in which translation has been arrested at a defined point in the nascent chain sequence, such that the NC remains covalently attached to the ribosome at the PTC. Translation arrest can be achieved by a range of methods, and the resulting RNCs can be purified for further study by a variety of structural, biochemical, or biophysical methods.
**Ribosome profiling**: method for determining the global distribution of ribosomes across the transcriptome, based on next generation sequencing of ribosome-protected mRNA fragments. Variations in ribosome density along a transcript can be used to determine translation rates with codon-specific resolution. Selective ribosome profiling is a refinement of this method, in which only ribosomes bound to a specific cofactor such as a molecular chaperone are isolated, and this can be used to map nascent chain–cofactor interactions on a global level.
**Synonymous mutation**: mutation in which, due to the degeneracy of the genetic code, the encoded amino acid is not changed. For example, UUU and UUC codons both encode phenylalanine.
